# Postural Control Characteristics in Alzheimer’s Disease, Dementia With Lewy Bodies, and Vascular Dementia

**DOI:** 10.1093/gerona/glae061

**Published:** 2024-02-27

**Authors:** Kosuke Fujita, Taiki Sugimoto, Hisashi Noma, Yujiro Kuroda, Nanae Matsumoto, Kazuaki Uchida, Yoshinobu Kishino, Takashi Sakurai

**Affiliations:** Department of Prevention and Care Science, Research Institute, National Center for Geriatrics and Gerontology, Morioka, Obu, Aichi, Japan; Japan Society for the Promotion of Science, Kojimachi, Chiyoda, Tokyo, Japan; Department of Prevention and Care Science, Research Institute, National Center for Geriatrics and Gerontology, Morioka, Obu, Aichi, Japan; Department of Data Science, Institute of Statistical Mathematics, Midori-cho, Tachikawa, Tokyo, Japan; Department of Prevention and Care Science, Research Institute, National Center for Geriatrics and Gerontology, Morioka, Obu, Aichi, Japan; Department of Prevention and Care Science, Research Institute, National Center for Geriatrics and Gerontology, Morioka, Obu, Aichi, Japan; Department of Prevention and Care Science, Research Institute, National Center for Geriatrics and Gerontology, Morioka, Obu, Aichi, Japan; Department of Rehabilitation Science, Kobe University Graduate School of Health Sciences, Tomogaoka, Suma, Kobe, Hyogo, Japan; Department of Prevention and Care Science, Research Institute, National Center for Geriatrics and Gerontology, Morioka, Obu, Aichi, Japan; Department of Cognition and Behavior Science, Nagoya University Graduate School of Medicine, Furo, Chikusa, Nagoya, Aichi, Japan; Department of Prevention and Care Science, Research Institute, National Center for Geriatrics and Gerontology, Morioka, Obu, Aichi, Japan; Department of Cognition and Behavior Science, Nagoya University Graduate School of Medicine, Furo, Chikusa, Nagoya, Aichi, Japan; (Medical Sciences Section)

**Keywords:** Balance, Digital biomarker, Memory clinic, Postural sway, Types of dementia

## Abstract

**Background:**

Dementia often results in postural control impairment, which could signify central nervous system dysfunction. However, no studies have compared postural control characteristics among various types of dementia. This study aimed to compare static postural control in patients with Alzheimer’s disease (AD), dementia with Lewy bodies (DLB), and vascular dementia (VaD).

**Methods:**

Cross-sectional relationship between the clinical diagnoses (AD, DLB, VaD, or normal cognition [NC]) of outpatients at a memory clinic and their upright postural control characteristics were examined. In the postural control test, participants were instructed to maintain a static upright standing on a stabilometer for 60 seconds under the eyes-open and eyes-closed conditions. Forty postural control parameters, including distance, position, and velocity in the anterior–posterior and medio–lateral directions, derived from the trajectory of the center of mass sway, were calculated. The characteristics of each type of dementia were compared to those of NC, and the differences among the 3 types of dementia were evaluated using linear regression models.

**Results:**

The study included 1 789 participants (1 206 with AD, 111 with DLB, 49 with VaD, and 423 with NC). Patients with AD exhibited distinct postural control characteristics, particularly in some distance and velocity parameters, only in the eyes-closed condition. Those with DLB exhibited features in the mean position in the anterior–posterior direction. In patients with VaD, significant differences were observed in most parameters, except the power spectrum.

**Conclusions:**

Patients with AD, DLB, and VaD display disease-specific postural control characteristics when compared to cognitively normal individuals.

Dementia is a major cause of loss of patients’ independence and of decreased quality of life. The total number of patients with dementia worldwide will triple in the next 3 decades (from 57 million in 2019 to an estimated 153 million in 2050) (1). Providing medical and formal or informal, long-term end-of-life care for patients with dementia poses a considerable strain on caregivers and is associated with a high financial cost, which has been estimated to bring the global economic burden of the disease to approximately $17 trillion in 2050 (2). Because the neuropsychiatric symptoms and sensorimotor dysfunction along with dementia are directly related to an increased caregiving load (3,4), the importance of appropriate assessment and management of these noncognitive symptoms of dementia has been pointed out (5,6).

In recent years, many studies have focused on gait and balance impairments in patients with dementia (7–10). Gait and balance deficits in patients with dementia are clinically significant issues because they are risk factors for negative outcomes such as activities of daily living (ADL) decline, falls, and dementia progression (11–13). Moreover, some studies imply that motor control measurements can be used as screening and diagnostic tools because gait and balance dysfunction are known to be already present at the mild cognitive impairment (MCI) or early dementia phase (8,14–16). Because adequate postural control requires the appropriate central nervous system (CNS) function (integration and coordination between inputs from somatosensory, visual, and vestibular systems and the outputs for postural muscles), quantitative measures of postural control characteristics are considered to reflect CNS performance (9,10). One established postural control measurement is the testing of random and consecutive movements of the center of gravity (postural sway) during quiet upright standing using a stabilometer. This measurement is a better comprehensive indicator of fall risk in older adults than clinical balance tests such as 1-leg stand and is commonly used in clinical practice in the fields of rehabilitation and otolaryngology (17). However, its adoption in the case of patients with dementia has been limited. Previous studies have shown that the magnitude and speed of postural sway increase with the onset of MCI and dementia (18,19). In addition, postural sway characteristics in older adults with dementia or cognitive impairment are also associated with their cognitive function (20), falls (17,19), volume or functional loss in specific brain regions (hippocampus, parahippocampal gyrus, entorhinal cortex, and inferior parietal lobe) (20–22), and greater default mode network functional connectivity at the resting state (23).

Dementia presents a variety of clinical and pathological features depending on its underlying causes (24). Since postural control requires a complex integration at the CNS level (25), it is possible that differences between different types of dementia, such as Alzheimer’s disease (AD), dementia with Lewy bodies (DLB), and vascular dementia (VaD), may have different effects on postural control characteristics. However, evidence with sufficient cases describing postural sway features in patients with dementia is limited, and the characteristics of postural control in different types of dementia have not been defined. The purpose of this study was to determine balance and postural control characteristics according to dementia type based on postural sway tests.

## Method

### Design, Settings, and Participants

This study examined cross-sectional differences in upright postural control among patients with AD, DLB, VaD, and those without clinical cognitive decline, based on a retrospective review of electronic medical records. First-time outpatients aged 65–85 years who visited the memory clinic of the National Center for Geriatrics and Gerontology (NCGG) between June 2011 and December 2017 were examined. According to our standard diagnostic procedure, patient and their family members (or primary caregivers) underwent a comprehensive geriatric assessment, blood testing, neuropsychological examination, and brain imaging at their initial visit. An experienced neurologist, psychiatrist, geriatrician, or neurosurgeon made a clinical diagnosis of normal cognition, MCI, or dementia (as well as specific dementia type) based on the results of these examinations and those from the medical interview (26). The National Institute on Aging and the Alzheimer’s Association Workgroup criteria, the fourth consensus report of the DLB Consortium, and the diagnostic criteria for vascular cognitive disorders by the International Society for Vascular Behavioral and Cognitive Disorders were used for the diagnosis of AD, DLB, and VaD, respectively (27–29). We have included in the analysis patients diagnosed with possible or probable AD, possible or probable DLB, or cortical/subcortical VaD (27–29). Patients who had visited the memory clinic during the study period due to a subjective cognitive concern but were diagnosed with normal cognition (NC) were also included in the analysis as a control group. Those who had missing values in the postural control test or MMSE were excluded. We also excluded participants with (a) limitations in basic ADLs (<80 points on the Barthel index); (b) diabetes mellitus (DM, self-reported, HbA1c > 6.5%, or use of anti-diabetic drugs); (c) other neurological or neurodegenerative diseases such as Huntington’s disease, normal pressure hydrocephalus, or multiple system atrophy; and (d) significant cerebrovascular disorders confirmed by brain imaging, to prevent potential confounding effects from musculoskeletal dysfunction and/or peripheral neuropathy.

The study protocol was created in accordance with the Helsinki Declaration and approved by the Ethics Committee of the NCGG (approval No. 1611). We provided an opt-out opportunity for those who did not consent to the use of their data in the study.

### Postural Control Measurement

The sway of the center of mass during upright standing was measured to assess postural control in the participants. The ground reaction force and center of pressure (CoP) were measured using a stabilometer―a force plate attached to a measuring device, Gravicorder GP-5000 (ANIMA Corporation, Japan), to estimate the center of mass sway. Trained psychologists conducted postural control testing in accordance with the Japanese equilibrium research guidelines (30). In a moderately bright and quiet examination room, participants were instructed to maintain an upright posture with their feet together and arms by the sides of the body for 60 seconds. Measurements were performed in the eyes-open and eyes-closed conditions, in that order. During the eyes-open condition, participants focused on a visual reference mark at a distance of 100 cm in front of them. The detailed test procedure is described elsewhere (30). Time-course trajectories of CoP were recorded as coordinates on a horizontal plane (with the x-axis representing the medio–lateral [ML] direction and the y-axis representing the anterior–posterior [AP] direction), with a sampling rate of 20 Hz. Of the 66 parameters validated in a previous study (31), we used 40 parameters (20 parameters per eye condition) from 5 domains (distance, area, position, velocity, and power spectrum) as outcome measures for the purpose of increasing interpretability. The details of each variable and calculation method are shown in Table 1. In the distance, area, and velocity domains, positive coefficient values indicated that participants with dementia moved their CoP faster and with a greater range than did participants with NC; in the position domain, positive coefficient values indicated that participants with dementia controlled their upright posture by keeping their CoP on the right side (for ML parameters) or front side (for AP parameters) more often than did individuals with NC; and in the power spectrum domain, positive coefficient values indicated that postural control in participants with dementia represents a larger proportion of the relevant frequency region than that in individuals with NC.

**Table 1.** Postural Sway Parameters Used in this Study

Domain	Parameter	Units	Short description
Distance	Mean path length	cm/s	CoP displacement per second.
	RMS	cm	Root mean square of CoP displacement. RMS = $\sqrt{\frac{1}{n}\underset{i=1}{\overset{n}{\sum }}\;\left\{{\left(xi-\bar{x}\right)}^{2}+{\left(yi-\bar{y}\right)}^{2}\right\}}$
	RMS (ML)	cm	RMS in x-axis. RMS (ML) = $\sqrt{\frac{1}{n}\underset{i=1}{\overset{n}{\sum }}\;{\left(xi-\bar{x}\right)}^{2}}$
	RMS (AP)	cm	RMS in y-axis. RMS (AP) = $\sqrt{\frac{1}{n}\underset{i=1}{\overset{n}{\sum }}\;{\left(yi-\bar{y}\right)}^{2}}$
Area	Rectangular area	cm^2^	The area enclosed by a rectangle with the maximum displacement in each axis.
	RMS area	cm^2^	Area enclosed by a circle with RMS as radius.
Position	Center of position (ML)	cm	Mean CoP coordinates in the x-axis.
	Center of position (AP)	cm	Mean CoP coordinates in the y-axis.
	SD of position (ML)	cm	Standard deviation of CoP coordinates in the x-axis.
	SD of position (AP)	cm	Standard deviation of CoP coordinates in the y-axis.
Velocity	Mean velocity (ML)	cm/s	Mean moving velocity of CoP in the x-axis.
	Mean velocity (AP)	cm/s	Mean moving velocity of CoP in the y-axis.
	SD of velocity (ML)	cm/s	Standard deviation of CoP moving velocity in the x-axis.
	SD of velocity (AP)	cm/s	Standard deviation of CoP moving velocity in the y-axis.
Power spectrum	Power of A-area (ML)	%	^*^Ratio of the x-axis signal at 0–0.2 Hz to the total power spectrum
	Power of B-area (ML)	%	^*^Ratio of the x-axis signal at 0.2–2 Hz to the total power spectrum
	Power of C-area (ML)	%	^*^Ratio of the x-axis signal at 2 Hz or over to the total power spectrum
	Power of A-area (AP)	%	^*^Ratio of the y-axis signal at 0–0.2 Hz to the total power spectrum
	Power of B-area (AP)	%	^*^Ratio of the y-axis signal at 0.2–2 Hz to the total power spectrum
	Power of C-area (AP)	%	^*^Ratio of the y-axis signal at 2 Hz or over to the total power spectrum

*Notes*: AP = anterior–posterior; CoP = center of pressure; ML = medio–lateral; RMS = root mean square.

Medio–lateral axis and anterior–posterior axis are denoted by x and y, respectively. *n* = number of data points (60 s × 20 Hz = 1 200); x*i*, yi = CoP coordinates in the x- and y-axis at each time. $\bar{x},\;\bar{y}$ = Mean CoP coordinates in the x- and y-axis.

^*^Fourier transform was performed on the CoP trajectories of the x- or y-axis.

### Demographic Data; Cognitive, Mental, and Physical Function; and Living and Medication Status

Demographic information such as age, sex, years of education, comorbidities (diabetes or neurodegenerative diseases), and pharmacological status were obtained through a questionnaire or medical interview. Global cognition was measured using the MMSE, with scores ranging from 0 to 30, and higher scores reflecting better cognitive performance (32). Depressive mood and basic ADLs were measured using the 15-item Geriatric Depression Scale (GDS) and Barthel index, respectively (33,34). We used body mass index (BMI) as a marker of nutritional status and maximal grip strength as a marker of physical function. Pharmacological status was represented in 2 ways: the number of medications, and the use of fall risk-increasing drugs (FRIDs). According to the definition of the European Geriatric Medicine Society Task, FRIDs include antihypertensives, antiarrhythmics, anticholinergics, antihistamines, sedatives, hypnotics, antipsychotics, antidepressants, opioids, and nonsteroidal anti-inflammatory drugs (35,36). Carrier status of apolipoprotein E (APOE) ε4 alleles was also included in the analysis as a dementia-related biomarker.

### Statistical Analyses

Clinical characteristics of the participants and raw postural control parameters are summarized as frequency (percentage) for categorical variables, mean ± standard deviation for normally distributed variables, or median [interquartile range] for non-normally distributed variables in each group. Postural control parameters were compared using a 1-way analysis of variance (ANOVA).

To examine the relationship between dementia type and postural control, we created a multi-variable linear regression model with postural control parameters as the response variables and dementia type as the explanatory variable. The effects of AD, DLB, and VaD on postural control parameters were estimated as coefficients with reference to NC, after adjusting for covariates (age, sex, years of education, number of medications, use of FRIDs, MMSE score, GDS score, Barthel index score, BMI, grip strength, and APOE status). Models were created for all of the 40 postural control parameters. Statistical significance was set at *p* < .05, and all statistical analyses were conducted using R software version 4.0.0 (R Foundation for Statistical Computing, Vienna, Austria).

To further examine the differences between dementia types regarding postural control parameters, we also performed direct comparisons between dementia types (AD vs DLB, AD vs VaD, and DLB vs VaD). Coefficients on postural control parameters for DLB and VaD with reference to AD, and for VaD with reference to DLB, were estimated using multivariate linear regression analysis with the covariates described above.

## Results

### Participant Characteristics Across Clinical Groups

**Figure 1.**
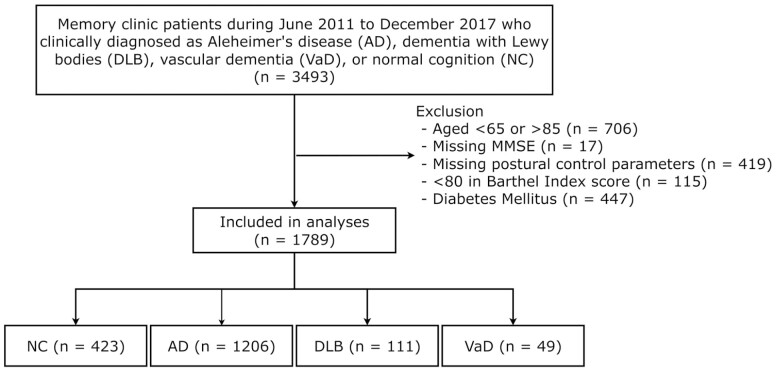
Study flowchart.

Among the patients who visited our hospital during the study period, 3 493 were diagnosed with NC, AD, DLB, and VaD. A total of 1 789 patients were included in the analysis after excluding patients aged <65 or >85 years (*n* = 706), lacking an MMSE score (*n* = 17) or postural control parameters (*n* = 19), with a Barthel index score <80 (*n* = 115), or with DM (*n* = 447; Figure 1). The analysis sample included 423 patients with NC, 1 206 with AD, 111 with DLB, and 49 with VaD. The average age was 76.5 years, and 33.3% were men (Table 2).

**Table 2.** Patients’ Baseline Clinical Characteristics

	Overall *N* = 1 789	NC *N* = 423	AD *N* = 1 206	DLB *N* = 111	VaD *N* = 49
Age, y (*n* = 1 789)	76.5 ± 5.3	73.3 ± 5.0	77.5 ± 5.1	77.7 ± 4.9	77.2 ± 5.1
Sex, male (*n* = 1 789)	595 (33.3%)	174 (41.1%)	356 (29.5%)	35 (31.5%)	30 (61.2%)
Education, y (*n* = 1 773)	11 [9, 12]	12 [10, 14]	9 [9, 12]	9 [9, 12]	9 [9, 12]
MMSE, score (*n* = 1 789)	21 [17, 25]	29 [27, 30]	19 [16, 22]	20 [16, 22]	20 [17, 23]
GDS, score (*n* = 1 784)	4 [2, 6]	3 [1, 5]	4 [2, 5]	5 [3, 7]	5 [2, 7]
Barthel index, score (*n* = 1 789)	100 [100, 100]	100 [100, 100]	100 [100, 100]	100 [95, 100]	95 [90, 100]
Body mass index, kg/m^2^ (*n* = 1 787)	21.8 ± 3.1	22.2 ± 3.0	21.6 ± 3.1	21.7 ± 3.5	22.9 ± 3.5
Grip strength, kg (*n* = 1 123)	20.8 ± 7.8	24.5 ± 8.6	19.6 ± 6.9	18.4 ± 7.9	19.8 ± 7.5
FRIDs use (*n* = 1 789)	442 (24.7%)	109 (25.8%)	273 (22.6%)	44 (39.6%)	16 (32.7%)
Drugs number, *n* (*n* = 1 789)	3 [1, 6]	3 [1, 6]	3 [1, 6]	5 [3, 7]	5 [3, 8]
APOE4 carrier (*n* = 1 154)	407 (35.3%)	46 (17.4%)	335 (43.0%)	21 (27.3%)	5 (15.2%)

*Notes*: AD = Alzheimer’s disease; APOE = apolipoprotein E; DLB = dementia with Lewy bodies; FRIDs = fall risk-increasing drugs; GDS = geriatric depression scale; MMSE = mini-mental state examination; VaD = vascular dementia.

Variables are presented as frequency (percentage) for categorical variables, as mean ± standard deviation for normally distributed variables, or as median [interquartile range] for non-normally distributed variables. The number of cases included in the analysis is listed next to the variable name.

### Association Between the Dementia Types and Postural Control Characteristics

The raw values of the postural sway parameters in each group are shown in Table 3 and Supplementary Figures 1–5. Almost all parameters in the distance and area domains―the distance or size of the area traveled by the CoP―and the velocity domain, which represents the speed of movement of the CoP, showed a tendency to increase in the eyes-open and eyes-closed conditions in the following order: NC < AD < DLB < VaD. The values of these parameters were greater in the eyes-closed condition than in the eyes-open condition (eg, eyes-open condition, root mean square: NC = 0.73, AD = 0.80, DLB = 0.89, VaD = 1.04; eyes-closed condition, root mean square: NC = 0.79, AD = 0.89, DLB = 0.94, VaD = 1.10). Regarding the position domain, no special characteristic was found in the ML side, but it was found in the AP side among the 4 groups in the eyes-open and eyes-closed conditions (in the eyes-open and eyes-closed conditions, ML, *P = *.596 and.505, respectively; AP, *P = *.016 and.038, respectively). In the power spectrum domain, dementia (especially DLB and VAD) exhibited a larger proportion of the low-frequency area (A-area) and a smaller proportion of the mid-frequency area (B-area) in the ML side.

**Table 3.** Patients’ Postural Sway Parameters

	Eyes-open condition					Eyes-closed condition				
	NC *N* = 423	AD *N* = 1 206	DLB *N* = 111	VaD *N* = 49	*p* Value	NC *N* = 423	AD *N* = 1 206	DLB *N* = 111	VaD *N* = 49	*p* Value
Mean path length, cm/s	1.51 ± 0.68	1.82 ± 0.76	1.92 ± 0.78	2.28 ± 1.24	<.001	2.11 ± 1.27	2.53 ± 1.41	2.45 ± 1.13	2.91 ± 1.59	<.001
RMS, cm	0.73 ± 0.20	0.80 ± 0.23	0.89 ± 0.28	1.04 ± 0.32	<.001	0.79 ± 0.27	0.89 ± 0.32	0.94 ± 0.32	1.10 ± 0.36	<.001
RMS (ML), cm	0.45 ± 0.15	0.53 ± 0.18	0.59 ± 0.22	0.69 ± 0.26	<.001	0.50 ± 0.20	0.59 ± 0.24	0.62 ± 0.25	0.74 ± 0.30	<.001
RMS (AP), cm	0.57 ± 0.18	0.58 ± 0.18	0.65 ± 0.23	0.76 ± 0.23	<.001	0.60 ± 0.22	0.66 ± 0.25	0.69 ± 0.24	0.80 ± 0.26	<.001
Rectangular area, cm^2^	8.76 ± 5.89	11.20 ± 8.03	13.99 ± 10.88	20.11 ± 14.96	<.001	12.46 ± 11.96	15.84 ± 13.67	17.72 ± 14.92	22.78 ± 18.77	<.001
RMS area, cm^2^	1.81 ± 1.10	2.15 ± 1.33	2.73 ± 1.97	3.70 ± 2.26	<.001	2.19 ± 1.82	2.82 ± 2.33	3.08 ± 2.26	4.18 ± 2.87	<.001
Center of position (ML), cm	0.11 ± 0.82	0.14 ± 0.95	0.01 ± 1.12	−0.05 ± 1.26	.596	0.01 ± 0.87	0.05 ± 0.99	0.03 ± 1.18	−0.21 ± 1.33	.505
Center of position (AP), cm	−0.84 ± 1.41	−0.75 ± 1.60	−1.22 ± 1.71	−0.99 ± 1.99	.016	−0.50 ± 1.45	−0.47 ± 1.62	−0.95 ± 1.76	−0.89 ± 2.01	.038
SD of position (ML), cm	0.45 ± 0.15	0.53 ± 0.18	0.59 ± 0.22	0.69 ± 0.26	<.001	0.50 ± 0.20	0.59 ± 0.24	0.62 ± 0.25	0.74 ± 0.30	<.001
SD of position (AP), cm	0.57 ± 0.18	0.58 ± 0.18	0.65 ± 0.23	0.76 ± 0.23	<.001	0.60 ± 0.22	0.66 ± 0.25	0.69 ± 0.24	0.80 ± 0.26	<.001
Mean velocity (ML), cm/s	0.83 ± 0.35	0.94 ± 0.38	1.00 ± 0.46	1.14 ± 0.55	<.001	1.13 ± 0.62	1.23 ± 0.65	1.19 ± 0.60	1.39 ± 0.77	.004
Mean velocity (AP), cm/s	0.83 ± 0.35	1.01 ± 0.41	1.07 ± 0.37	1.32 ± 0.68	<.001	1.13 ± 0.63	1.40 ± 0.74	1.37 ± 0.57	1.68 ± 0.85	<.001
*SD* of velocity (ML), cm/s	1.38 ± 0.58	1.56 ± 0.64	1.68 ± 0.78	1.91 ± 0.94	<.001	1.89 ± 1.04	2.07 ± 1.10	1.99 ± 1.02	2.34 ± 1.28	.003
*SD* of velocity (AP), cm/s	1.38 ± 0.59	1.68 ± 0.70	1.77 ± 0.63	2.18 ± 1.12	<.001	1.87 ± 1.05	2.32 ± 1.21	2.29 ± 0.97	2.81 ± 1.38	<.001
Power of A-area (ML), %	25.68 ± 6.32	26.37 ± 7.34	26.78 ± 7.46	27.40 ± 7.81	.288	22.02 ± 6.22	22.65 ± 7.20	24.62 ± 7.41	24.13 ± 9.10	.011
Power of B-area (ML), %	60.02 ± 6.39	59.09 ± 6.73	58.76 ± 7.61	57.99 ± 7.41	.059	62.08 ± 6.55	60.88 ± 6.83	60.15 ± 7.75	60.58 ± 8.62	.011
Power of C-area (ML), %	14.30 ± 4.18	14.54 ± 4.38	14.46 ± 4.65	14.61 ± 4.31	.820	15.90 ± 4.50	16.48 ± 5.05	15.23 ± 4.54	15.29 ± 5.41	.027
Power of A-area (AP), %	30.48 ± 7.03	26.54 ± 7.45	27.32 ± 7.45	27.65 ± 7.77	<.001	24.01 ± 6.35	21.69 ± 7.14	23.09 ± 7.83	23.04 ± 8.03	<.001
Power of B-area (AP), %	52.86 ± 6.40	54.88 ± 6.74	54.37 ± 7.01	53.92 ± 6.09	<.001	57.57 ± 6.25	57.52 ± 6.70	56.40 ± 8.30	57.67 ± 6.68	.946
Power of C-area (AP), %	16.66 ± 5.29	18.58 ± 5.76	18.31 ± 6.21	18.43 ± 6.29	<.001	18.42 ± 5.55	20.79 ± 6.54	20.51 ± 6.31	19.30 ± 6.56	<.001

*Notes*: AD, Alzheimer’s disease; AP, anterior–posterior; APOE, apolipoprotein E; DLB, dementia with Lewy bodies; ML, medio–lateral; RMS, root mean square; *SD*, standard deviation; VaD, vascular dementia.

All variables are presented as mean ± standard deviation. The *p* values calculated by 1-way analysis of variance are presented.

The adjusted odds ratio of the effects of each dementia type (in comparison with NC) on postural control parameters calculated using a linear regression model are shown in Table 4. Overall, participants with dementia presented with a greater range, displacement, and velocity of CoP movement than did those with NC, after adjustment for covariates. Postural control parameters in AD participants were not significantly different from those in the NC group in the eyes-open condition. In contrast, in the eyes-closed condition, AD participants were found to have significantly different postural control characteristics compared to those in individuals with NC, except for the position of the center of position and some of the power spectrum parameters: (coefficient [95% confidence interval] Mean path length = 0.43 [0.13 to 0.72]; RMS = 0.12 [0.05 to 0.20]; Rectangular area = 5.00 [1.92 to 8.08]; the values for all the parameters analyzed are shown in Table 4). DLB and VaD participants also showed different postural control characteristics compared to those shown by individuals with NC in several parameters, although there were no marked differences between the eyes-open and eyes-closed conditions. DLB was characterized by a standing position with a posterior CoP position; DLB participants kept their center of mass backward (ie, stooped posture), especially in the eyes-open condition, as demonstrated by the results corresponding to the center of CoP position (AP) (coefficient [95% CI], −0.60 [−1.16 to −0.05]). Among the 3 types of dementia in this study, only VaD did not affect the power spectrum parameters: (eg, coefficient [95% confidence interval] Power of A-area (ML) in eyes-open condition = 1.88 [−1.56 to 5.32]; Power of A-area (ML) in eyes-closed condition = 0.40 [−2.95 to 3.75]).

**Table 4.** Effect of Dementia on the Postural Sway Parameters Estimated by Multi Variable Linear Regression

	Eyes-open condition			Eyes-closed condition		
	AD (ref. NC)	DLB (ref. NC)	VaD (ref. NC)	AD (ref. NC)	DLB (ref. NC)	VaD (ref. NC)
Mean path length	0.06 [−0.11 to 0.22]	0.21 [−0.03 to 0.45]	**0.42 [0.10 to 0.73]**	**0.43 [0.13 to 0.72]**	0.27 [−0.17 to 0.71]	0.43 [−0.14 to 1.01]
RMS	0.00 [−0.05 to 0.06]	**0.14 [0.06 to 0.23]**	**0.21 [0.10 to 0.31]**	**0.12 [0.05 to 0.20]**	**0.22 [0.10 to 0.33]**	**0.28 [0.13 to 0.43]**
RMS (ML)	0.02 [−0.02 to 0.07]	**0.13 [0.07 to 0.20]**	**0.18 [0.09 to 0.26]**	**0.09 [0.03 to 0.15]**	**0.17 [0.09 to 0.26]**	**0.23 [0.12 to 0.34]**
RMS (AP)	−0.01 [−0.06 to 0.03]	**0.08 [0.01 to 0.15]**	**0.12 [0.03 to 0.21]**	**0.08 [0.02 to 0.14]**	**0.14 [0.04 to 0.23]**	**0.18 [0.06 to 0.30]**
Rectangular area	0.20 [−1.72 to 2.11]	**4.32 [1.50 to 7.14]**	**8.08 [4.38 to 11.79]**	**5.00 [1.92 to 8.08]**	**8.74 [4.20 to 13.28]**	**8.93 [2.96 to 14.89]**
RMS area	0.00 [−0.32 to 0.33]	**0.88 [0.40 to 1.35]**	**1.35 [0.73 to 1.98]**	**0.88 [0.31 to 1.44]**	**1.51 [0.67 to 2.34]**	**1.88 [0.78 to 2.98]**
Center of position (ML)	0.02 [−0.22 to 0.25]	−0.07 [−0.41 to 0.28]	0.23 [−0.23 to 0.69]	0.07 [−0.18 to 0.32]	0.22 [−0.15 to 0.59]	0.33 [−0.15 to 0.82]
Center of position (AP)	−0.08 [−0.45 to 0.30]	−**0.60 [**−**1.16 to** −**0.05]**	−0.17 [−0.89 to 0.56]	−0.09 [−0.49 to 0.30]	−0.49 [−1.07 to 0.10]	−0.26 [−1.02 to 0.51]
*SD* of position (ML)	0.02 [−0.02 to 0.07]	**0.13 [0.07 to 0.20]**	**0.18 [0.09 to 0.26]**	**0.09 [0.03 to 0.15]**	**0.17 [0.09 to 0.26]**	**0.23 [0.12 to 0.34]**
*SD* of position (AP)	−0.01 [−0.06 to 0.03]	**0.08 [0.01 to 0.15]**	**0.12 [0.03 to 0.21]**	**0.08 [0.02 to 0.14]**	**0.14 [0.04 to 0.23]**	**0.18 [0.06 to 0.30]**
Mean velocity (ML)	0.02 [−0.07 to 0.11]	**0.18 [0.04 to 0.31]**	**0.24 [0.07 to 0.41]**	**0.19 [0.04 to 0.34]**	**0.22 [0.00 to 0.44]**	**0.32 [0.03 to 0.61]**
Mean velocity (AP)	0.04 [−0.05 to 0.12]	0.10 [−0.03 to 0.23]	**0.27 [0.11 to 0.44]**	**0.25 [0.10 to 0.40]**	0.15 [−0.08 to 0.37]	**0.29 [0.00 to 0.59]**
*SD* of velocity (ML)	0.03 [−0.12 to 0.18]	**0.30 [0.08 to 0.52]**	**0.41 [0.12 to 0.70]**	**0.34 [0.09 to 0.60]**	0.36 [−0.01 to 0.74]	**0.55 [0.06 to 1.05]**
*SD* of velocity (AP)	0.06 [−0.08 to 0.21]	0.16 [−0.05 to 0.38]	**0.47 [0.19 to 0.76]**	**0.42 [0.16 to 0.67]**	0.26 [−0.12 to 0.63]	**0.51 [0.02 to 1.00]**
Power of A-area (ML)	1.21 [−0.56 to 2.99]	1.61 [−1.01 to 4.23]	1.88 [−1.56 to 5.32]	−0.37 [−2.10 to 1.36]	**3.68 [1.13 to 6.23]**	0.40 [−2.95 to 3.75]
Power of B-area (ML)	−1.80 [−3.52 to −0.08]	−1.57 [−4.11 to 0.97]	−1.94 [−5.28 to 1.40]	−1.09 [−2.82 to 0.65]	−**2.77 [**−**5.32 to** −**0.21]**	−0.32 [−3.68 to 3.04]
Power of C-area (ML)	0.58 [−0.51 to 1.67]	−0.04 [−1.64 to 1.57]	0.06 [−2.05 to 2.17]	**1.45 [0.22 to 2.69]**	−0.91 [−2.73 to 0.91]	−0.08 [−2.48 to 2.31]
Power of A-area (AP)	−1.56 [−3.29 to 0.18]	−0.56 [−3.11 to 1.99]	−0.42 [−3.78 to 2.93]	−0.70 [−2.45 to 1.05]	1.57 [−1.02 to 4.15]	1.39 [−2.00 to 4.79]
Power of B-area (AP)	1.13 [−0.51 to 2.77]	0.35 [−2.07 to 2.76]	1.97 [−1.20 to 5.15]	−0.02 [−1.71 to 1.66]	−0.98 [−3.46 to 1.50]	1.48 [−1.79 to 4.74]
Power of C-area (AP)	0.43 [−0.96 to 1.81]	0.21 [−1.83 to 2.26]	−1.55 [−4.23 to 1.14]	0.72 [−0.79 to 2.24]	−0.59 [−2.82 to 1.64]	−2.87 [−5.81 to 0.06]

*Notes*: AD = Alzheimer’s disease; AP = anterior–posterior; DLB = dementia with Lewy bodies; ML = medio–lateral; RMS = root mean square; *SD* = standard deviation; VaD = vascular dementia.

The effects of each type of dementia for postural sway parameters, relative to normal cognition are presented as estimated coefficient (95% confidence interval). Bold type represents statistical significance (*p* < .05).

The adjusted odds ratio from the direct comparison between the 3 dementia types is shown in Supplementary Table 1. Compared to that of AD, DLB, and VaD had significantly larger values for many parameters such as root mean square and standard deviation of position. This characteristic was also observed in the eyes-closed condition, but it was more prominent in the eyes-open condition. In contrast, when comparing DLB and VaD, none of the parameters, except for standard deviation of velocity (AP) in the eyes-open condition were significantly different. Parameters in the power spectrum domain were found to have fewer items with differences than those in other domains (Supplementary Table 1).

## Discussion

In this study, we examined the differences in postural control characteristics among 3 types of dementia. The results revealed that patients with different types of dementia exhibit different postural control strategies. Patients with AD were characterized by differences between the eyes-open and eyes-closed condition. Those with DLB exhibited a distinctive posture in which their center of mass was positioned backward. Those with VaD showed greater postural sway values compared to patients affected by the other 2 types of dementia in the distance, area, and velocity domains. However, none of the parameters in the power spectrum domain presented significant differences.

Overall, patients with dementia sustained their upright posture by displacing their CoP to a greater extent compared to individuals with normal cognition, which agrees with the results of previous studies (7,16,18–21). However, some of the results for individual parameters were inconsistent with those from previous studies. Deschamps et al. concluded that MCI or mild to moderate AD affects the velocity domain more than the distance/length domain of postural control parameters (18). In contrast, in our study, dementia affected all these domains (distance, length, and velocity domains) similarly. For instance, AD did not affect any of these parameters compared with patients with NC in the test with the eyes-open condition; In the test with the eyes-closed condition, however, AD affected all these parameters. Although the reason for the difference between the results of the current study and those of Deschamps et al. is unclear, methodological differences may have contributed. They used age and BMI as matching variables but did not consider other factors that affect postural sway parameters. We obtained more robust results by using a larger sample size and adjusting for multiple covariates including ADL function, mental function, and medication status that might mediate postural control parameters. However, further validation is warranted, especially in considering racial differences such as body size.

Previous studies of healthy older adults suggest that poorer balance associated with normal aging may be due to a functional decline in the CNS (ie, central postural control systems) rather than of the peripheral sensorimotor system (37). Postural control modification in patients with dementia could also reflect the pathological modification in the CNS. This study confirmed that patients with dementia have increased postural sway, even when we restricted the analysis to patients without peripheral neuropathy or muscle weakness that would interfere with ADLs. This highlights the hypothesis that changes in postural control in patients with dementia also reflect pathological changes in the central nervous system.

Compared to individuals with NC, patients with AD did not present significant differences in postural control in the eyes-open condition; however, they showed differences in postural control in the eyes-closed condition, which suggests that CNS dysfunction caused by AD might attenuate the postural adjustment response that follows blinding. When the eyes are closed, standing balance is maintained by increasing reliance on the vestibular sensory and somatosensory systems. However, in patients with AD, the center of mass motion may be increased owing to inadequate compensation as a result of impaired integration of sensory information in the postural center systems. Although the mechanism underlying the impaired postural control in patients with dementia is not sufficiently understood yet, some previous studies have shown potential associations between postural control and the volume of the basal ganglia (eg, nucleus accumbens and putamen), as well as with the volume and neurometabolite levels in areas related to memory function such as the hippocampus (20–22). Moreover, decreased acetylcholine levels in the brain due to AD may negatively affect postural control because the pedunculopontine tegmental nucleus, which is rich in cholinergic neurons, plays an important role in postural control (38–40). Analyses focusing on the association between postural control and structural/functional connectivity (such as the default mode network) or investigations on the effects of dementia at the brainstem level may reveal new aspects of AD in the future.

The result that patients with DLB kept their center of mass backward may partially reflect abnormal postural reflexes in Parkinsonism. The pull test may become an effective physical assessment tool in clinical practice in cases where it is difficult to distinguish DLB from other dementia disorders (41,42). Patients with VaD showed significantly different postural control characteristics compared to those shown by NC individuals in a wide number of parameters. They also exhibited more evident changes in the comparison between the 3 types of dementia. Because regional periventricular hyperintensity and deep white matter hyperintensity in patients with AD are associated with postural sway parameters (14), the current finding in patients with VaD may reflect the effects of diffuse white matter lesions on various brain regions. The association between postural control and brain imaging, especially in patients with VaD, is an important topic for future investigation.

Previous studies have examined motor control in patients with dementia, particularly focusing on gait (43–48). Gait characteristics are represented by several parameters, such as stride length and cadence. It is becoming clear that variability, rather than the pace or rhythm, is a more important domain associated with dementia and cognitive function (47). Increased variability in step length or time (reduced symmetry or consistency) may partially reflect abnormalities in postural control. The difference between maintaining an upright posture and gait is that the former involves static postural control while the latter involves dynamic postural control. However, because few studies have compared static and dynamic postural control in patients with dementia, the effect of these postural control strategies on the diagnosis, management, and treatment of dementia has not been properly described. Further research is needed to confirm the difference between static and dynamic motor control strategies in order to establish a motor control-based dementia diagnosis/classification method in the future.

The results of this study are relatively robust because we analyzed a large sample of patients compared to that in previous studies (18,21); however, there are several limitations regarding the study design. First, external validity was limited because this was a single-center study with no validation cohort. Further investigations targeting not only the clinical setting but also various populations are needed to verify the applicability of postural control-based diagnosis and classification of dementia in the future. Second, as participants in the control group were individuals who visited the memory clinic with subjective cognitive concerns, their health status was uncertain, which could have led to biased results. Further comparisons with healthy older adults living in the community are required to obtain more robust results. Third, because we focused on 20 highly interpretable parameters (40 parameters if the eyes-open and eyes-closed conditions are considered separately) in this study, not all parameters of CoP trajectory testing that have been validated in previous studies were used. Quijoux et al. identified 66 potentially reliable parameters (31). Moreover, based on the idea that the high complexity of the CoP trajectory reflects the ability to adapt to environmental modifications, mathematical approaches such as chaos analysis (7,31) have been considered in recent years. Finally, we did not strictly specify the presence of peripheral neuropathy, which could have potentially affected the results of this study. On the other hand, considering that diabetes is a major cause of peripheral neuropathy in middle-aged and older adults (49), the potential confounding effect of peripheral neuropathy would have been minimized in this study because participants with diabetes were excluded from the analysis.

In conclusion, different types of dementia have different postural controls. The results of this study provide insight into the mechanisms underlying the balance and gait dysfunction in patients with dementia, which are particularly important in dementia care. Moreover, the findings also present the possibility of postural control testing as a non-invasive and simple approach for investigating central nervous system abnormalities related to dementia.

## Supplementary Material

glae061_suppl_Supplementary_Figures_S1-S5_Table_S1
