# Supplementary material for: Postural Control Characteristics in Alzheimer’s Disease, Dementia With Lewy Bodies, and Vascular Dementia
Source: J Gerontol A Biol Sci Med Sci. 2024 Feb 27;79(4):glae061. doi: 10.1093/gerona/glae061 (PMC10949438; doi:10.1093/gerona/glae061)
Supplement: glae061_suppl_Supplementary_Figures_S1-S5_Table_S1 [file glae061_suppl_supplementary_figures_s1-s5_table_s1.docx]

**Supplemental Contents**

**eFigure1. – eFigure5.** Postural sway parameters discriminated according to the type of dementia

**eTable1.** Inter-dementia type differences in the effect of postural sway parameters estimated by multivariable linear regression

**eFigure1. Postural sway parameters discriminated according to the type of dementia**

**
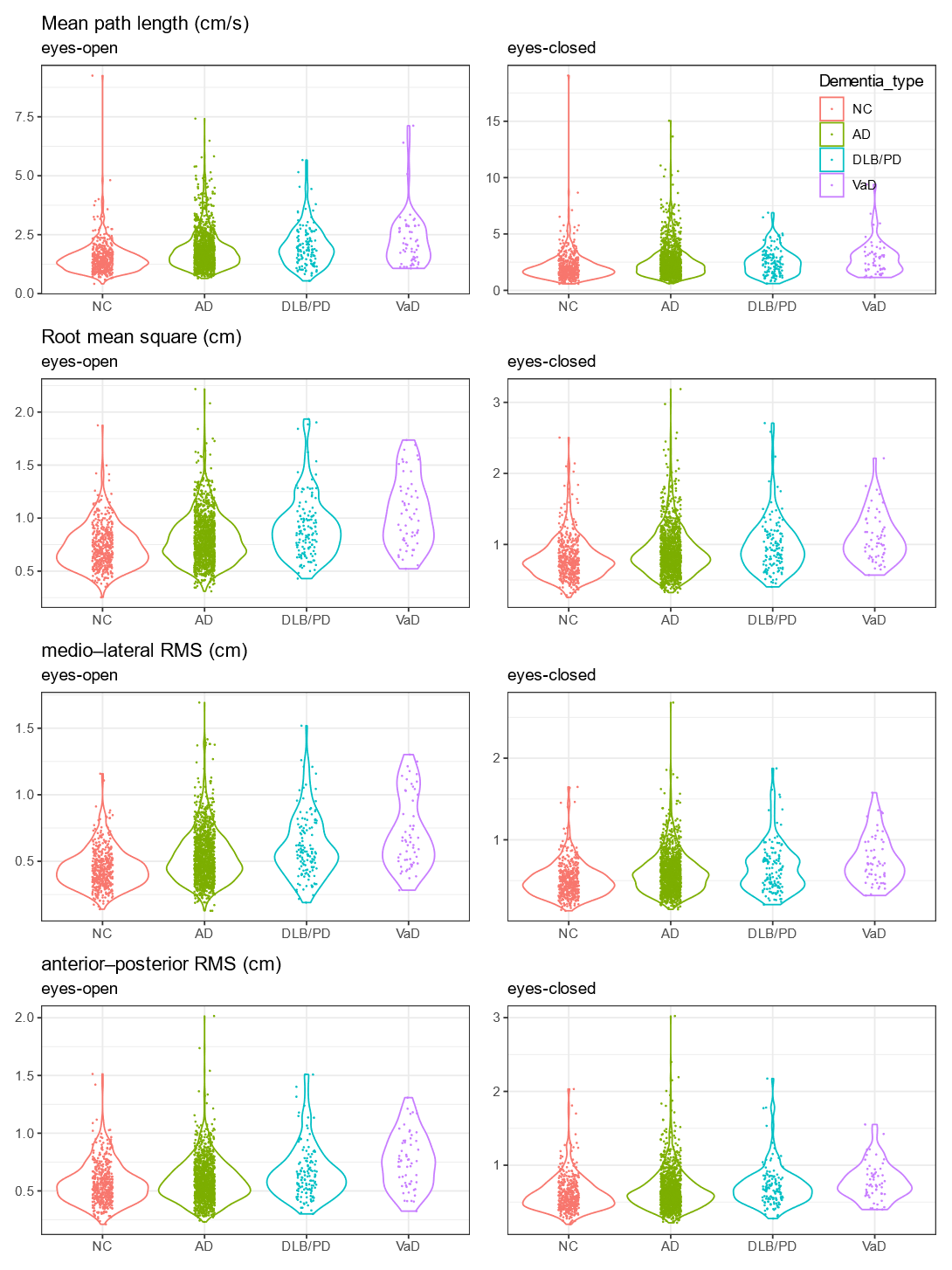
**

**eFigure2. Postural sway parameters discriminated according to the type of dementia (continued)**

**
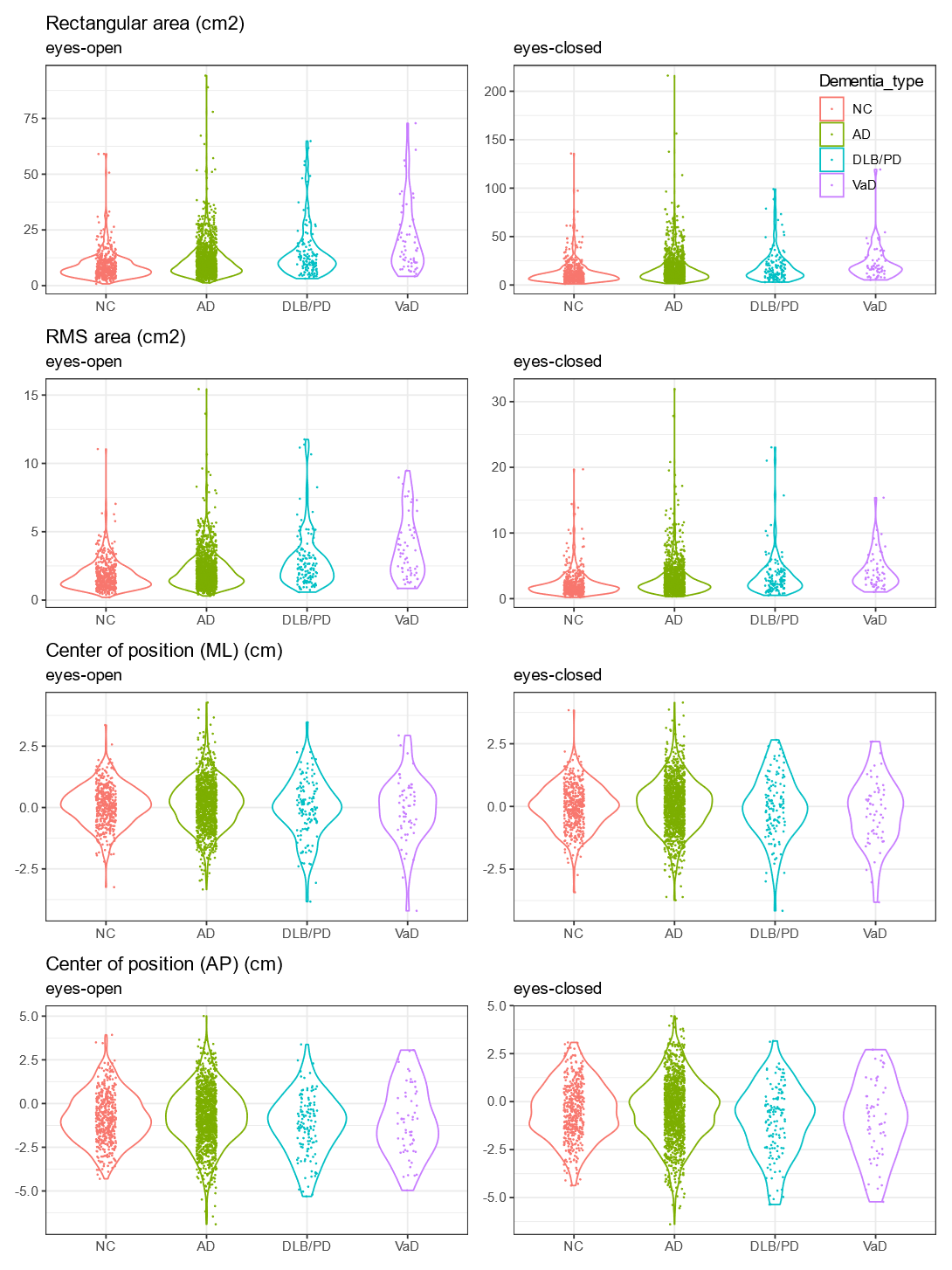
**

**eFigure3. Postural sway parameters discriminated according to the type of dementia (continued)**

**
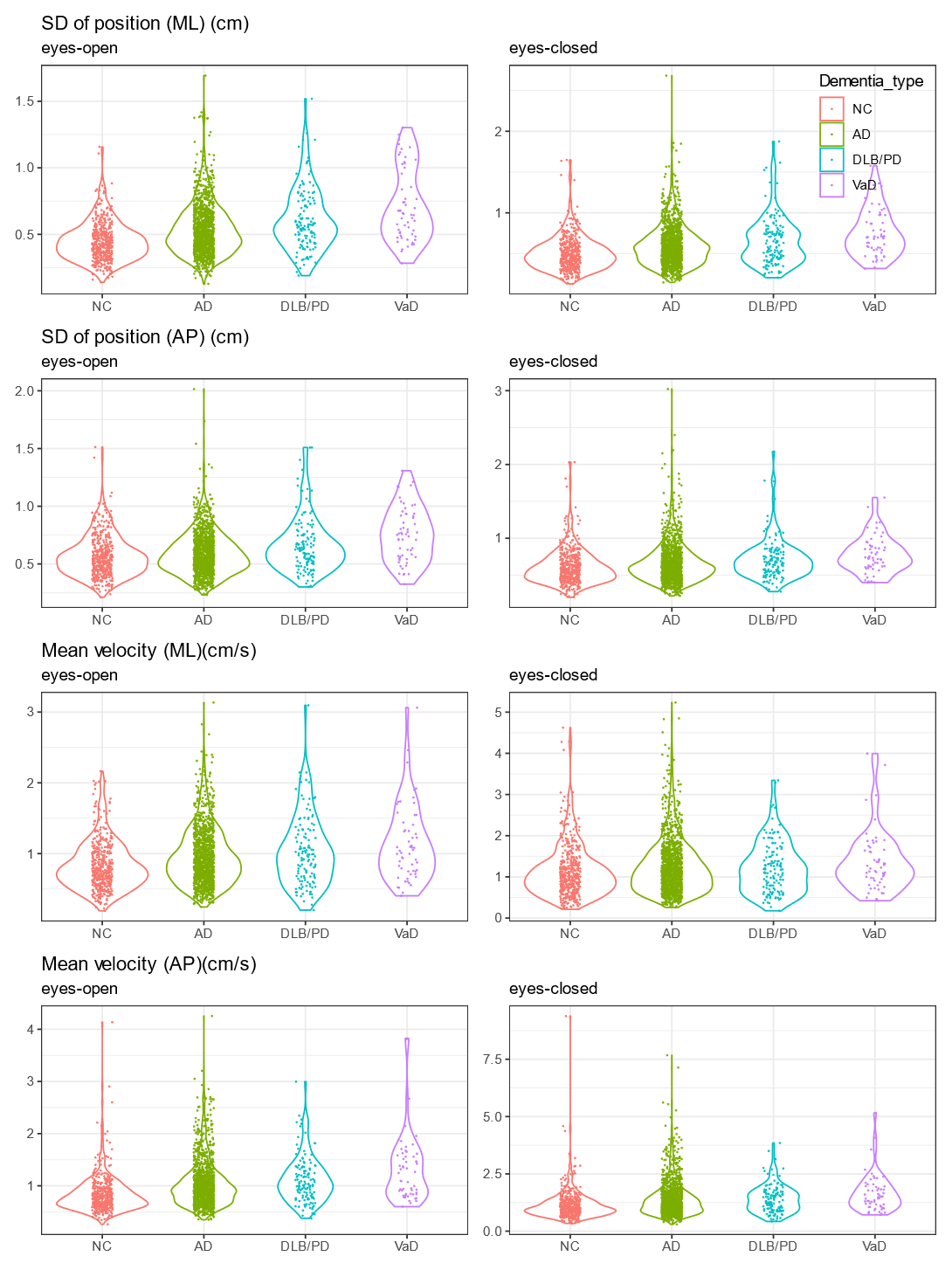
**

**eFigure4. Postural sway parameters discriminated according to the type of dementia (continued)**

**
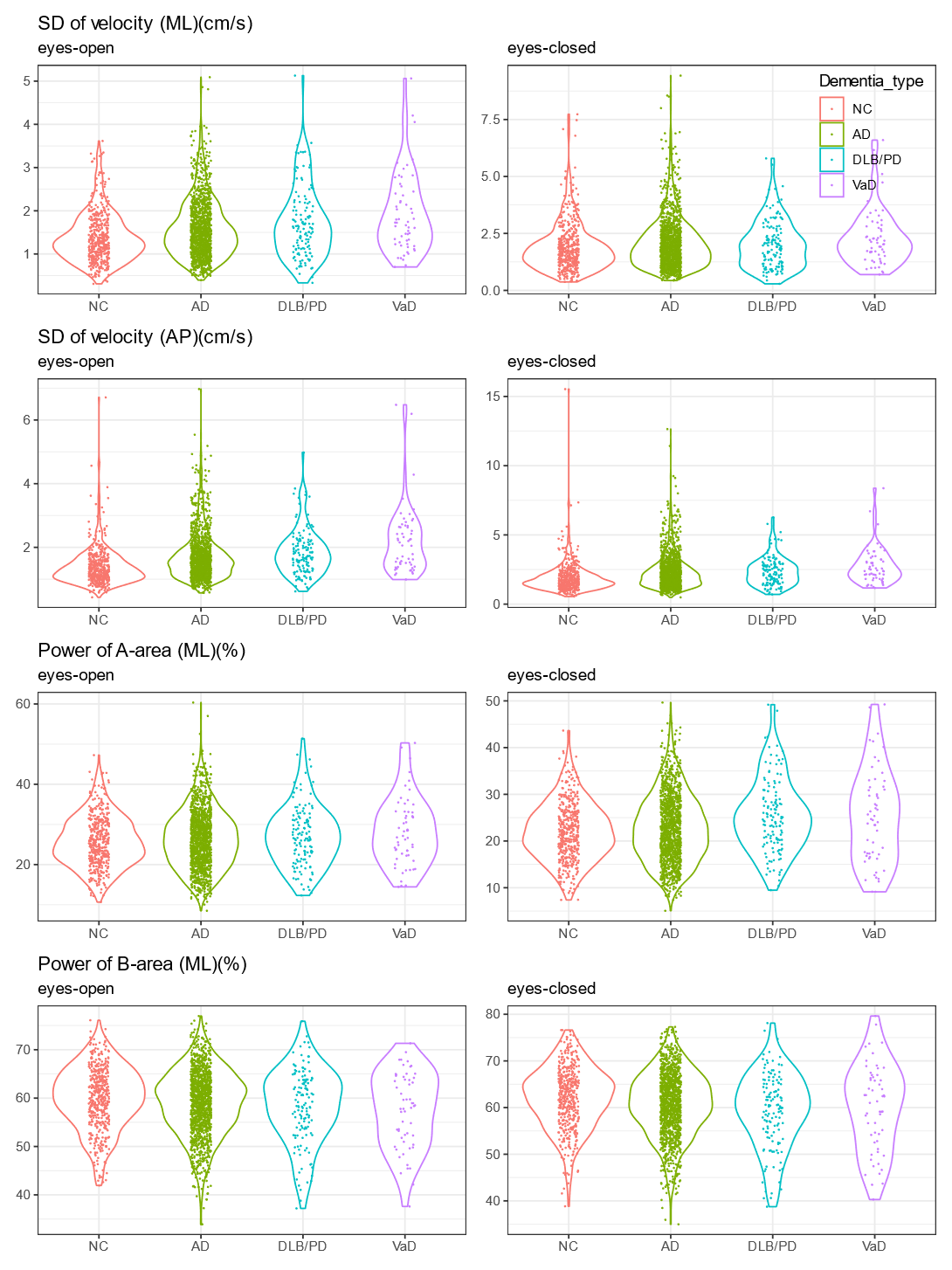
**

**eFigure5. Postural sway parameters discriminated according to the type of dementia (continued)**

**
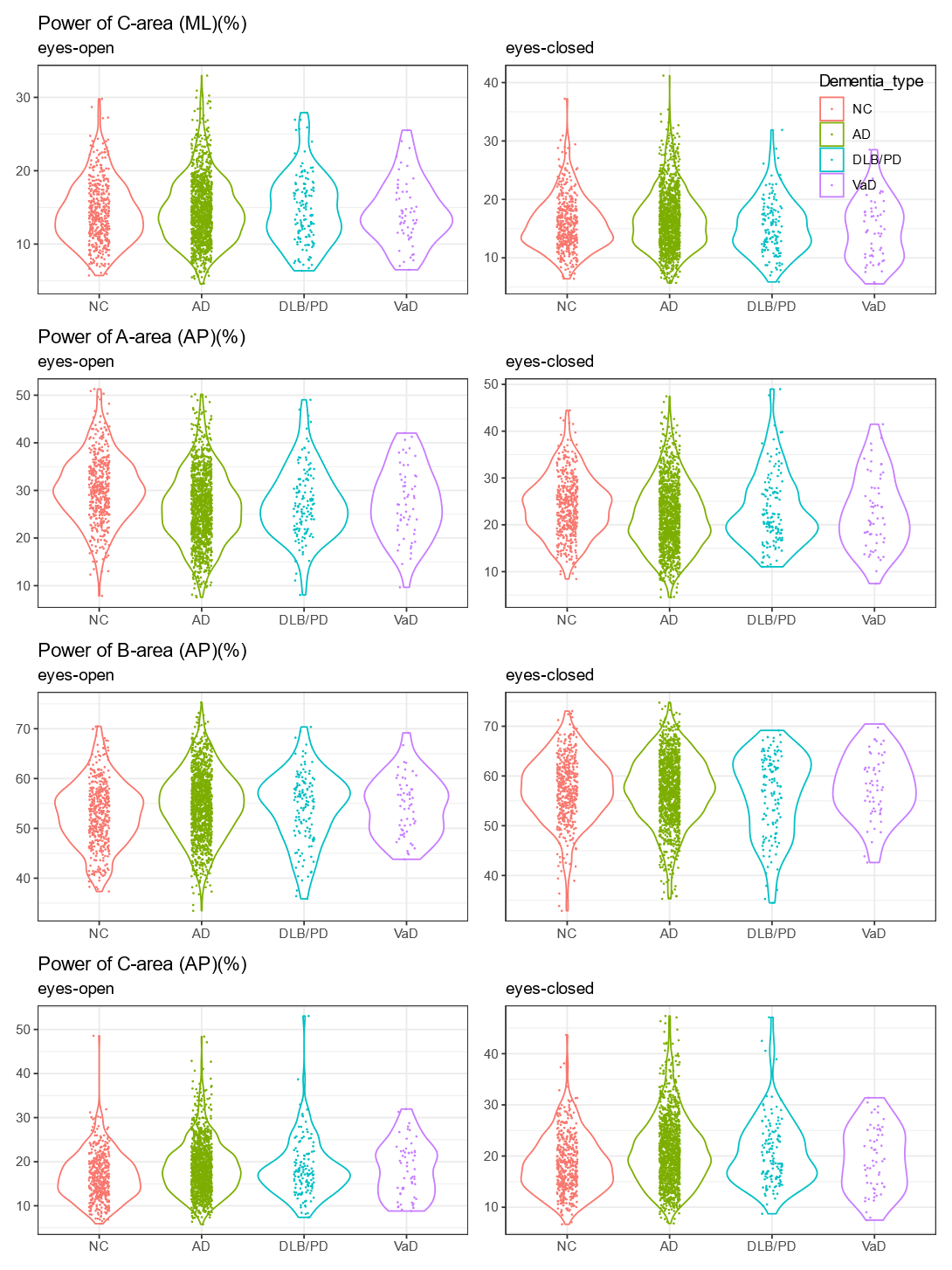
**

**eTable1. Inter-dementia type differences in the effect of postural sway parameters estimated by multivariable linear regression**

|  | **Eyes-open condition** | | |  | **Eyes-closed condition** | | |
| --- | --- | --- | --- | --- | --- | --- | --- |
|  | **DLB (ref: AD)** | **VaD (ref: AD)** | **VaD (ref: DLB)** |  | **DLB (ref: AD)** | **VaD (ref: AD)** | **VaD (ref: DLB)** |
| Mean path length | 0.16 [ -0.05 to 0.36] | **0.36 [ 0.07 to 0.65]** | 0.20 [ -0.13 to 0.54] |  | -0.16 [ -0.53 to 0.21] | 0.01 [ -0.53 to 0.54] | 0.16 [ -0.45 to 0.78] |
| RMS | **0.14 [ 0.07 to 0.21]** | **0.20 [ 0.10 to 0.30]** | 0.06 [ -0.05 to 0.18] |  | **0.09 [ 0.00 to 0.19]** | **0.16 [ 0.02 to 0.30]** | 0.07 [ -0.09 to 0.23] |
| RMS (ML) | **0.11 [ 0.06 to 0.16]** | **0.15 [ 0.08 to 0.23]** | 0.04 [ -0.05 to 0.13] |  | **0.08 [ 0.01 to 0.15]** | **0.14 [ 0.04 to 0.24]** | 0.06 [ -0.06 to 0.18] |
| RMS (AP) | **0.09 [ 0.03 to 0.15]** | **0.13 [ 0.05 to 0.22]** | 0.04 [ -0.05 to 0.14] |  | 0.05 [ -0.02 to 0.13] | 0.10 [ -0.01 to 0.21] | 0.04 [ -0.09 to 0.17] |
| Rectangular area | **4.12 [ 1.74 to 6.51]** | **7.89 [ 4.45 to 11.33]** | 3.76 [ -0.22 to 7.75] |  | 3.74 [ -0.10 to 7.59] | 3.93 [ -1.61 to 9.47] | 0.19 [ -6.22 to 6.60] |
| RMS area | **0.87 [ 0.47 to 1.28]** | **1.35 [ 0.77 to 1.93]** | 0.48 [ -0.20 to 1.15] |  | 0.63 [ -0.08 to 1.34] | 1.00 [ -0.02 to 2.02] | 0.37 [ -0.81 to 1.55] |
| Center of position (ML) | -0.08 [ -0.38 to 0.21] | 0.21 [ -0.21 to 0.64] | 0.30 [ -0.20 to 0.79] |  | 0.15 [ -0.17 to 0.46] | 0.26 [ -0.19 to 0.71] | 0.12 [ -0.41 to 0.64] |
| Center of position (AP) | **-0.53 [ -0.99 to -0.06]** | -0.09 [ -0.77 to 0.58] | 0.44 [ -0.34 to 1.22] |  | -0.39 [ -0.88 to 0.10] | -0.16 [ -0.87 to 0.55] | 0.23 [ -0.59 to 1.05] |
| SD of position (ML) | **0.11 [ 0.06 to 0.16]** | **0.15 [ 0.08 to 0.23]** | 0.04 [ -0.05 to 0.13] |  | **0.08 [ 0.01 to 0.15]** | **0.14 [ 0.04 to 0.24]** | 0.06 [ -0.06 to 0.18] |
| SD of position (AP) | **0.09 [ 0.03 to 0.15]** | **0.13 [ 0.05 to 0.22]** | 0.04 [ -0.05 to 0.14] |  | 0.05 [ -0.02 to 0.13] | 0.10 [ -0.01 to 0.21] | 0.04 [ -0.09 to 0.17] |
| Mean velocity (ML) | **0.16 [ 0.04 to 0.27]** | **0.22 [ 0.06 to 0.38]** | 0.07 [ -0.12 to 0.25] |  | 0.02 [ -0.16 to 0.21] | 0.13 [ -0.14 to 0.40] | 0.11 [ -0.21 to 0.42] |
| Mean velocity (AP) | 0.06 [ -0.05 to 0.17] | **0.24 [ 0.08 to 0.39]** | 0.18 [ -0.01 to 0.36] |  | -0.10 [ -0.29 to 0.09] | 0.04 [ -0.23 to 0.32] | 0.14 [ -0.17 to 0.46] |
| SD of velocity (ML) | **0.27 [ 0.08 to 0.45]** | **0.38 [ 0.11 to 0.65]** | 0.11 [ -0.20 to 0.43] |  | 0.02 [ -0.30 to 0.33] | 0.21 [ -0.25 to 0.67] | 0.19 [ -0.34 to 0.72] |
| SD of velocity (AP) | 0.10 [ -0.08 to 0.29] | **0.41 [ 0.14 to 0.68]** | **0.31 [ 0.00 to 0.62]** |  | -0.16 [ -0.48 to 0.16] | 0.09 [ -0.36 to 0.55] | 0.25 [ -0.28 to 0.78] |
| Power of A-area (ML) | 0.40 [ -1.82 to 2.61] | 0.67 [ -2.53 to 3.86] | 0.27 [ -3.43 to 3.97] |  | **4.05 [ 1.89 to 6.21]** | 0.77 [ -2.34 to 3.88] | -3.28 [ -6.88 to 0.32] |
| Power of B-area (ML) | 0.22 [ -1.93 to 2.37] | -0.14 [ -3.24 to 2.96] | -0.37 [ -3.95 to 3.22] |  | -1.68 [ -3.85 to 0.48] | 0.77 [ -2.35 to 3.89] | 2.45 [ -1.16 to 6.06] |
| Power of C-area (ML) | -0.62 [ -1.98 to 0.74] | -0.52 [ -2.49 to 1.44] | 0.10 [ -2.18 to 2.37] |  | **-2.37 [ -3.91 to -0.82]** | -1.54 [ -3.76 to 0.69] | 0.83 [ -1.74 to 3.40] |
| Power of A-area (AP) | 0.99 [ -1.17 to 3.16] | 1.13 [ -1.98 to 4.25] | 0.14 [ -3.47 to 3.74] |  | **2.27 [ 0.08 to 4.46]** | 2.09 [ -1.06 to 5.25] | -0.17 [ -3.83 to 3.48] |
| Power of B-area (AP) | -0.78 [ -2.83 to 1.26] | 0.84 [ -2.10 to 3.79] | 1.63 [ -1.78 to 5.04] |  | -0.96 [ -3.06 to 1.15] | 1.50 [ -1.53 to 4.53] | 2.46 [ -1.05 to 5.97] |
| Power of C-area (AP) | -0.21 [ -1.94 to 1.52] | -1.98 [ -4.47 to 0.52] | -1.76 [ -4.65 to 1.12] |  | -1.31 [ -3.20 to 0.58] | **-3.59 [ -6.32 to -0.87]** | -2.28 [ -5.44 to 0.87] |

NOTE. The effects of each type of dementia for postural sway parameters, relative to other types of dementia are presented as estimated coefficient (95% confidence interval). Bold type represents statistical significance (p < 0.05). Abbreviations: AD, Alzheimer’s disease; AP, Anterior-posterior side; DLB, Dementia with Lewy bodies; ML, Mediolateral side; RMS, Root mean square; SD, standard deviation; VaD, Vascular dementia
